# Social Stability Risk Assessment of Land Expropriation: Lessons from the Chinese Case

**DOI:** 10.3390/ijerph16203952

**Published:** 2019-10-17

**Authors:** Chenxi Li, Zenglei Xi

**Affiliations:** 1 School of Public Administration, Xi’an University of Architecture and Technology, Xi’an 710055, China; lichenxi@xauat.edu.cn; 2 School of Economics, Hebei University, Baoding 071000, China; 3 Department of Geography, University of Wisconsin–Madison, Madison, Wisconsin, WI 53706, USA

**Keywords:** social stability risk, land expropriation, risk assessment, Chinese land expropriation construction project

## Abstract

Scholars have paid much attention to the problems existing in the land expropriation risk assessment system and the sound countermeasures from a qualitative perspective. Empirical research on land expropriation social stability risk assessment from the micro-level perspective is limited. This study analyzed the Chinese social stability risk assessment system of land expropriation though a case study of a land expropriation project in China. The current social stability risk assessment system of land expropriation, which includes the assessment purposes, principles, contents, methods, and results, was analyzed. We concluded with lessons and deficiencies from the current social stability risk assessment system. The research findings show that: (1) the current land expropriation risk assessment system mostly takes the land administration department as the main body of responsibility, identifies the risks by means of seminars, visits, letters, and visits, and takes the opinion of the masses or experts as the risk assessment result. (2) The current land expropriation risk assessment system should be standardized in terms of defining the risk assessment of land expropriation, improving the land expropriation risk assessment system and optimizing land expropriation assessment procedures. This paper provides a reference for the sustainable development of land use in rural and urban areas in China.

## 1. Introduction

Many developing countries worldwide have undergone very rapid urbanization in recent decades [1,2]. With this rapid development of urbanization, a large amount of agricultural land has been expropriated as infrastructure and national construction land, which has greatly met the needs of social development. However, there are many problems in the process of land expropriation that have negative impacts on social stability and development. Therefore, the problem of land acquisition conflict urgently needs to be solved.

The urbanization rate increased from only 17.9% in 1978 to 52.6% in 2012, and in March of 2014 in China, the central government put forward an ambitious plan to realize an urbanization rate of 60% by 2020 [3,4]. By promoting more peasants to migrate into urban areas, the process of urbanization fulfills the government’s responsibility to allow more people to enjoy the advantages and benefits of rapid development. During urbanization, ironically, there have been various risks and uncertainties in environmental, economic, and social aspects, bringing severe damage to people’s (especially peasants’) welfare and well-being [5,6,7,8,9]. Accompanying the rapid urbanization, numerous infrastructures, especially engineering construction projects and modern industrial construction projects, have been extensively constructed, unavoidably requiring the support of land expropriation and land acquisition [10,11]. The peasantry and rural residents are always the vulnerable groups, having to leave their original places of residence and losing the agricultural land where they have lived for many years [12]. All over the world, there has been an agreement that the peasants should receive compensation during agricultural land conversion [13,14,15,16]. Although citizens should give land priority to public and national interests, the landowners in developed countries do not necessarily incorporate their own incentives in the political decisions of land conversion [17], at which time compensation is paid based on the market value standard, and the planned construction projects easily move forward [13].

Different from land ownership in other countries, all land and associated natural resources in China are public property, where the agricultural land is collectively owned. Peasants and rural residents are given the right to agricultural land under contract, but they cannot directly pursue its market value through land exchange [13]. This means that the peasants cannot enjoy the incremental value the land gains from construction projects after land expropriation, while the local governments and developers become the biggest beneficiaries [13]. Nevertheless, the undisclosed compensation payment process is another significant problem that has undermined the amount of compensation peasants eventually receives [12]. Peasants have no political bargaining power in determining their compensation; rather, the country and township government and the village administration decide the income of land sales [12,13]. It is therefore pointed out that a large percentage of profits are detained by the collective, and peasants can receive very low compensation [18]. Meanwhile, although the central government anticipates that peasants will still be able to enjoy the profits of modern industries and employment opportunities, there are still many challenges at the local government level that deter the rural population from being the primary beneficiaries [19]. Subsequently, some landless peasants who have to migrate into cities for jobs cannot enjoy the social welfare and security because of the Hukou system [20], and sometimes, their inherent agricultural behaviors are discriminated against by original city dwellers [21]. The Hukou system is a population management system implemented by the Chinese government to the citizens of the People’s Republic of China in mainland China. According to the relationship between the region and family members, the Hukou system divides the household registration into “agricultural hukou” (agricultural household registration) and “non-agricultural hukou” (urban household registration). Agricultural hukou can allow households to obtain the right to use land in the countryside but cannot affect some policies in the city [22]. Household registration shows the legitimacy of natural persons living in a certain place. Therefore, land expropriation implies that landless peasants lose their last economic shelter if they cannot adapt to off-farm jobs [23,24].

Inadequate land compensation and uncertain future employment opportunities drive the occurrence of expropriation violence. For instance, it is reported that there were 17,900 cases of massive rural incidents in the first three quarters of 2006, and 385,000 peasants had actual conflicts with the government [25]. Unfortunately, the local governments concentrated more on economic development instead of peasants’ social welfare and wellbeing, as they conducted forcible and violent expropriation of peasants’ land [26]. The violence has not only become an obstacle hindering the progress of urbanization but has also been a trigger of societal instability [27]. Therefore, the central government has reformed and legitimated that local governments have to conduct social risk management prior to infrastructure construction. This is because social risk management has been regarded as an effective approach to predicting, analyzing, and determining the factors that may affect social stability before major matters are carried out (policy decisions, major engineering construction projects, major activities, and construction of important infrastructure facilities, etc.), thereby formulating, issuing, and implementing risk response strategies, plans, and measures to prevent, reduce, and eliminate risks [28,29,30].

In the context of China, although the central government has drawn lessons from other countries and reformed several times to produce socially friendly approaches, the local government may implement them in unsound patterns. Therefore, to determine the practical implications of social risk management, various studies on the implementation of social risk management have been carried out. For instance, in terms of hydraulic infrastructure construction projects, the scope and approaches of risk management were introduced [31]. Based on a case study of the Jixian Industrial Park project, a local government-driven key economic development project, the pattern of how local governments implement social risk management was revealed. This study primarily illustrated the framework used to conduct social risk management of large construction projects, determining that the processes of land expropriation and acquisition cannot be investigated clearly. Based on Yueqing City and Jiaxing City, Zhejiang Province, the variations of possible factors leading to conflicts in land acquisition with background conditions were identified [32]. However, the whole process and the effectiveness of conducting social risk management in land expropriation cannot be understood. Therefore, this paper aims to reveal the framework of social risk management in land expropriation and to further assess the effectiveness of social risk management after land expropriation. The study is carried out in the context of a Jingsong Old-age Care Services Center in Dingzhou City, a pilot city for the latest land expropriation and acquisition policy. The examination of the effectiveness of social risk management is not only useful for identifying the problems in social risk management, but it is also meaningful to further drive the socially friendly reform of land expropriation and acquisition. Specifically, the reminder of this paper is structured as follows: Section 2 overviews the evolution of land expropriation policies in China, the causes of land expropriation conflicts, land expropriation conflict management, and social stability risk assessment. Section 3 introduces the case study area and the framework of social risk management that will be utilized for land expropriation. Section 4 rates the social stability risks in land expropriation and further assesses the effectiveness of social risk management after land expropriation. Section 5 discusses the drawbacks and problems in the implementation of social risk management in land expropriation, and Section 6 concludes this paper and proposes some recommendations for the next reform step.

## 2. Literature Review

With the frequent occurrence of land expropriation conflicts in recent years, scholars have paid a significant amount of attention to land expropriation issue [33]. How to solve the conflict of land expropriation has also become a hot research topic [34]. We review research on the evolution of land expropriation policies, causes of land expropriation conflicts, land expropriation conflict management, and social stability risk assessment caused by land expropriation in China.

### 2.1. The Evolution of Land Expropriation Policies in China

Land problems related to political stability and societal security have been significant concerning since ancient Chinese times. Since the foundation of the People’s Republic of China, the government has undertaken several stages of land expropriation reform. In 2004, in particular, the central government announced that land acquisition or expropriation should be implemented for the sake of the public interest [35]. Although it is widely acknowledged that there are several differences between land acquisition and expropriation, Chinese laws do not distinguish between land acquisition and expropriation but refer to all land acquisition behavior by the government as land expropriation [36,37]. Therefore, this section adopts the terminology of “land expropriation” to present the evolution of land appropriation policies in China.

The progress is divided into five stages: (1) The establishment of land expropriation (1950–1957). The framework for land expropriation in China was preliminarily established. (2) The stagnation of land expropriation (1958–1981)—after the “Cultural Revolution” began in 1966, the government’s work was completely affected [38]. Land expropriation was at a standstill until the Third Plenary Session of the Eleventh Central Committee [39]. (3) The intensification of land expropriation management (1982–1996)—this stage moved land management in China from chaos to governance, and land management was strengthened. Nevertheless, there were many commanding characteristics in land expropriation. As a result, the basic means of land expropriation was hierarchical quota examination and approval, and the planning control was not sufficient, which affected land expropriation [40]. (4) A combination of land expropriation and land transfer occurred (1997–2011). At this stage, land expropriation was explored and perfected in accordance with the requirements of establishing a socialist market economy system. The most prominent characteristic was that land use control was carried out according to the overall land use plan [41]. However, because the planning system was not perfect, there was some weakness in the implementation of the plan. For example, local governments modified and broke through their plans at will [42]. (5) The reform of land expropriation (2011–current)—after 2008, the reform of the land expropriation system received extensive attention after the Chinese government put forward the “reform of the land expropriation system” [9]. In 2011, the Ministry of Land and Resources approved 11 cities for land expropriation projects. The pilot project involved reducing the scope of land expropriation, reforming the examination and approval system, perfecting the compensation and placement of land expropriation, and implementing social security for famers who had been expropriated. Nevertheless, the pilot work faced great difficulties in reducing the scope of land expropriation and reforming the examination and approval system. After 2016, The Ministry of Land and Resources began to try to jointly reform the pilot areas and combine the “three plots reform” [43]. Since 2015, 33 counties and cities have launched “three pilot reform” (including rural land expropriation reform, the profitable collectively-owned construction land entering the market, homestead reform) projects for rural land reform [43]. Nevertheless, the duration of the three reforms was too short, and the results achieved had some limitations and could not be widely spread and applied throughout the country [44].

Based on the evolution of land expropriation in China mentioned above, land expropriation was greatly influenced by the social and economic system [45]. In the transformation from private ownership of land to collective ownership of land, and from a highly centralized planned economy to a socialist market economic system, land expropriation has undergone significant changes. Land expropriation was a basic system of land use in China. The change in land expropriation reflected the change in the basic land use policy, especially through the protection of cultivated land and the influence of the situation of saving land. Therefore, land expropriation reform should be regarded as a systemic reform rather than an individual reform [46]. At different stages of development, the main contradictions faced by the land expropriation system in implementation were different. For example, in the era of planned economy, although the level of compensation for land expropriation was very low, the land expropriation and resettlement methods included employment arrangements [47]. Therefore, land expropriation was generally welcomed by farmers. Land expropriation was not difficult, but it led to excessive land waste [39]. Since the reform and opening up, although the compensation standard for land expropriation has been raised repeatedly, it has become increasingly difficult to obtain land due to the cancellation of employment arrangements [36]. Obviously, this problem cannot be solved simply by raising the compensation standard for land expropriation or by narrowing the scope of land expropriation. Therefore, land expropriation reform needs to find appropriate entry points and focus points according to the changes in major contradictions in different periods, and it should be improved along with household registration, employment, social security, and collective property rights.

### 2.2. Causes of Land Expropriation Conflicts

At present, research on the causes of land expropriation conflict mainly focus on land conflict from the point of view of land systems, property rights, social and economic development imbalance, and land expropriation compensation.

Land system reform is the direct result of important changes in the relations of the production of a society, which break the original patterns of land occupation and distribution [48]. However, the groups of interest in the original land system with damaged interests try their best to restore their original interests by means of resistance or even struggle. Under this situation, the difficulty of land system reform increases, and there is a hidden danger for the emergence of land conflict [49]. The ambiguity of land rights regards the inducement of all kinds of land conflict [50]. The unclear ownership of land leads to confusion about the right to use land. The final result of the competition for the right to use land resources is land conflict; this is also an important factor that induces land conflict [51,52]. The causes of land conflict also include an imbalance in socio-economic development [53,54,55]. Land conflict may be also caused by a contradiction between fixed land resources and a growing population, between foreign population migration and the original land use mode, and between land price change and land production potential [56]. In addition, the contradiction between low land expropriation compensation and the rising land price leads to farmers being in an inferior position [57]. Tagliarino et al. proposed that negative socioeconomic impacts are caused by land acquisition without adequate compensation, and they thought a transparent and participatory process should be fulfilled during land expropriation and community compensation [33]. As a result, due to dissatisfaction with land expropriation compensation, there is conflict between farmers and land expropriators [58,59].

### 2.3. Land Expropriation Conflict Management

The purpose of studying land expropriation conflict is to realize the effective management of social stability risk caused by land expropriation conflict [60]. The settlement of land expropriation conflicts is considered to be a process, for example, Petrescu-Mag et al. proposed that it is a process of management, circumvention, transformation, and resolution. Moreover, in this process, the importance of transformation is significantly higher than that of the other three, because transformation will make the possible land expropriation conflict develop in a favorable direction [61]. In China, research on the conflict management of land expropriation has obvious regional characteristics. The management of land expropriation conflict is generally considered from the perspective of social stability and economic interest. From the perspective of maintaining social stability, as an important part of farmers’ rights protection activities, farmers’ land disputes have threatened social stability [62]. From the perspective of economic interests, landless farmers should get reasonable compensation [63].

### 2.4. Social Stability Risk Assessment

According to social risk theory, it is difficult to eliminate the risk completely when it comes into being, but the risk manager can effectively avoid the harm of risk by actively controlling it [64]. At the end of the 1980s, Chinese scholars began to study the risk assessment of social stability. In 2012, the government stipulated that social stability risk assessment should be carried out for major fixed asset investment projects. Social stability assessment was mainly carried out using the social risk assessment index system and the social risk assessment method.

Regarding the social risk assessment index system, on the basis of comparative analysis and summary of the laws, regulations, and technical standards of social stability risk assessment of housing expropriation in various places, combined with the characteristics of housing expropriation, Cai and Zhang established a risk assessment model for the social stability of housing expropriation from legal, reasonable, feasible, and safety aspects, and selected the effective analytic hierarchy process (AHP) and blur comprehensive assessment methods by using AHP and fuzzy comprehensive evaluation methods synthetically [65]. Liu et al. proposed a practical framework of social risk management to identify specific social risks under the dominance of the local government [10].

Regarding the social risk assessment method, Liu and Li put forward the framework for social stability risk assessment (SSRA) in China by using theoretical and normative methods from the point of view of the social risk of a national engineering project. The results of the study provided a clear functional explanation for the formation mechanism and evolution model of social stability risk assessment, and a systematic technical evaluation was carried out [66].

Through the above analysis of research on social stability risk assessment, it can be observed that there is a lack of theoretical research on the factors and mechanisms influencing social stability risk, the identification method of social stability risk, and the evaluation method of the social stability risk grade, especially regarding social stability risk assessment caused by land expropriation.

## 3. Methodology

### 3.1. Study Context

**Figure 1:**
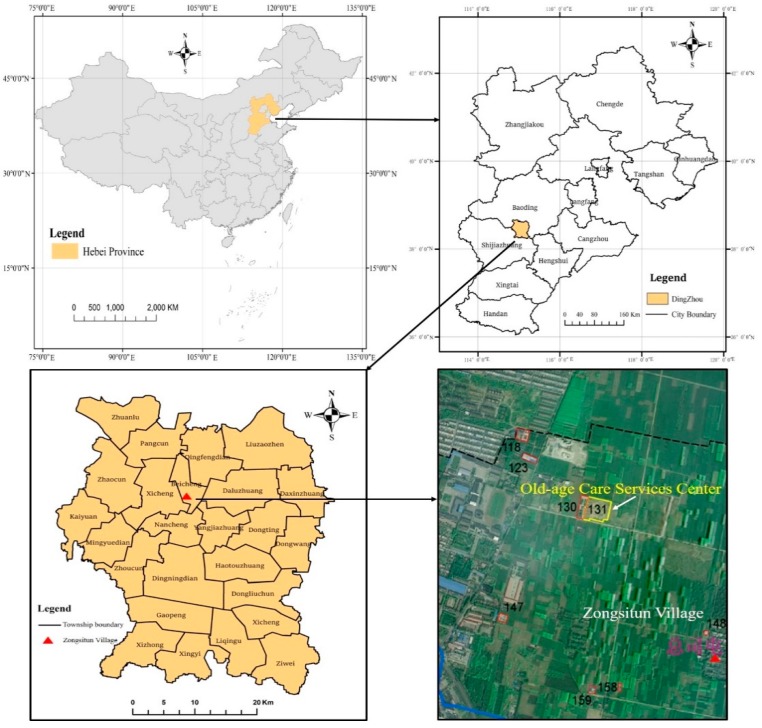
Location of the Jingsong Old-age Care Services Center.

The land expropriation project under study is being carried out in the Jingsong Old-age Care Services Center in Dingzhou City, Hebei Province, China. Jinsong Old-age Care Services Center has been levied 4.97 hectares of collective land in Zongsitun Village, Beicheng District. The project is a non-profit pension institution and is used for medical and health charity land. The land is transferred and negotiated by agreement. The price of the land parcel was 52,364.81 USD in 2016. The land will be levied as state-owned land with the approval of the Hebei provincial government in the form of the tenth batch of land for construction in 2016. The planned land use will be for social welfare. The plot is located in north of Dingcheng Village, Beicheng District, Dingzhou City, Hebei Province (Figure 1).

### 3.2. Framework of Social Risk Management for Land Expropriation

Through the social stability risk assessment, we aimed to achieve the following: identification of the potential risks of the land expropriation project and the risk level; the development of risk prevention measures; avoidance, reduction, or control of social stability risks that may arise within the scope of this project through dealing with them at the source; safeguarding of the implementation of the project and the interests of citizens, legal persons, and other organizations; prevention and dissolution of any mass social conflicts and disputes that may be caused by the parties involved in the implementation of the project; the maintenance of social stability so as to improve the construction project plan; and improvement of the project’s decision-making and approval process.

**Figure 2:**
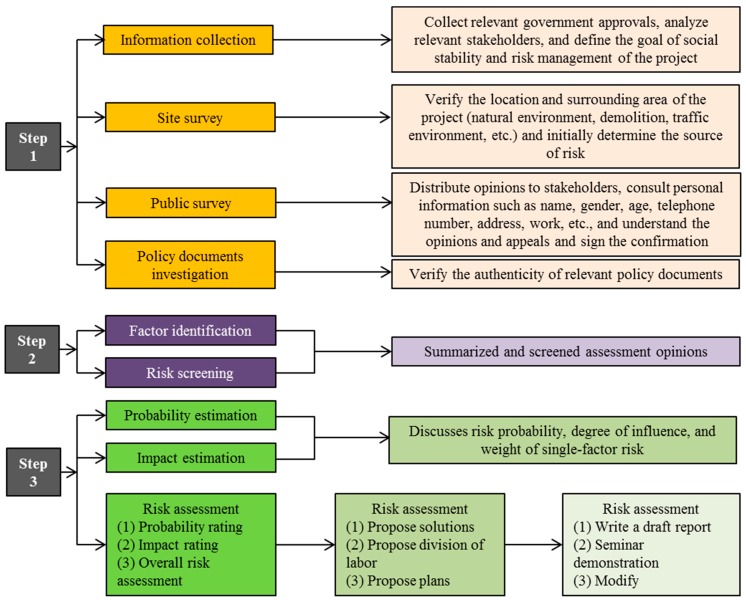
Framework of social risk management for land expropriation.

The assessment framework mainly includes site survey, public survey, and comprehensive analysis methods. In a “site survey”, the social stability risk assessment group verifies the authenticity of the project. Specifically, the social stability risk assessment group verifies the location and surrounding area of the project (natural environment, demolition, traffic environment, etc.) and initially determines the sources of risk. In a “public survey”, the social stability risk assessment group investigates the reflections of land-expropriated farmers on the land acquisition project. Specifically, the social stability risk assessment group distributes opinions to stakeholders; records personal information such as name, gender, age, telephone number, address, work, etc.; and aims to understand the opinions and appeals and sign the confirmation for the land acquisition project (Figure 2).

#### 3.2.1. Assessment Purposes

Through the social stability risk assessment, we aimed to achieve the following: identification of the potential risks of the land expropriation project and the risk level; the development of risk prevention measures; avoidance, reduction, or control of social stability risks that may arise within the scope of this project through dealing with them at the source; safeguarding of the implementation of the project and the interests of citizens, legal persons, and other organizations; prevention and dissolution of any mass social conflicts and disputes that may be caused by the parties involved in the implementation of the project; the maintenance of social stability so as to improve the construction project plan; and improvement of the project’s decision-making and approval process.

#### 3.2.2. Assessment Contents

(1) Validity Analysis and Assessment

- Is it in line with the policies of the country and is it is in conflict with the existing laws, regulations, or policies?
- Are the definitions of stakeholders involved accurate? Is the justification of the adjustment valid?
- Is it in compliance with the provisions of the decision-making processes or procedures?

(2) Reasonable Analysis and Assessment

- Is it in line with the law of economic development?
- Does it take the interests of the people into account?
- Is it compatible with the affordability of the community?

(3) Feasibility analysis and assessment

Is it compatible with the level of local economic and social development? Does the project implementation have the corresponding manpower, material resources, and financial resources? Have the related supporting measures been thoroughly and scientifically demonstrated, and are the timing and conditions for the promulgation ripe?

(4) Controllability analysis and assessment

- Has there been a public announcement on potential safety hazards? Will it lead to mass incidents, petitions, and negative public issues?
- Does the risk of social stability have the appropriate preventive and resolving measures? Are the measures effective or not?

#### 3.2.3. Assessment Methods

(1) Public Survey

**Figure 3:**
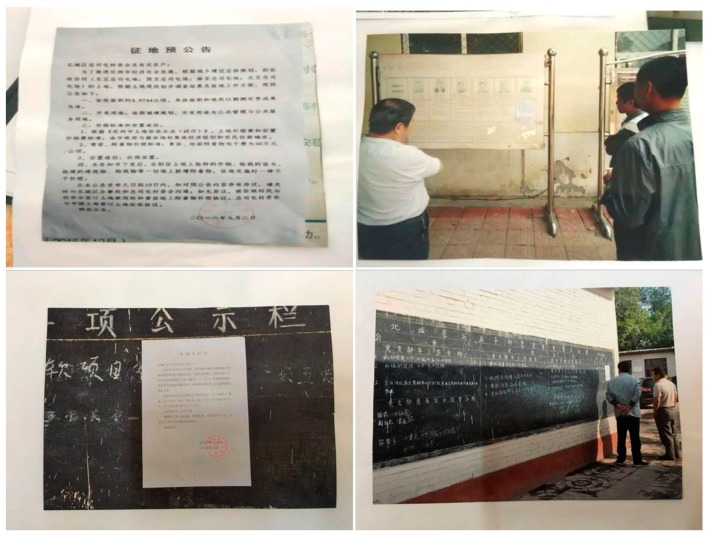
The announcement of land expropriation in the column of Zongsitun Village.

From August 20, 2016 to September 2, 2016, the Dingzhou Land Resources Bureau released the Announcement of Land expropriation in the column of Zongsitun Village, informing land requisitioned villages and relevant farmers of the intended use of land, compensation rates, production and living arrangements, and social security measures (Figure 3).

(2) Questionnaire

We conducted a questionnaire survey on the famers who had been expropriated. A total of 92 questionnaires were distributed, 91 were recovered, and there were 89 valid questionnaires; thus, the effective rate was 96.74%. Through field research at the headquarters of Tunchun Village in Beicheng Ddistrict, interviews with villager representatives, heads of village collectives, and persons in charge of the land expropriation reform of government departments were conducted. Meanwhile, questionnaire surveys among farmers were conducted (Table 1). It was found that a land expropriation pilot project had been successfully carried out. In the process of land expropriation, compensation and resettlement for land expropriation were more scientific and rational than before, and the villagers were more satisfied with the land expropriation work. The work of the village collective staff members was better promoted. In order to make the villagers understand, the government issued a series of support policies and documents. These all contributed to the further implementation and improvement of the pilot land expropriation reform in Dingzhou City.

#### 3.2.4. Controllability Assessment

(1) Risk Identification

The factors that led to social stability risks in the general land expropriation project were mainly due to compensation for land expropriation and house demolition in accordance with the *Measures for Land expropriation in Dingzhou City* (for trial implementation) [67]. According to the features of the expropriated land and the surrounding environment, the characteristics of the project were initially identified (Table 2).

(2) Initial Risk Level

**Figure 4:**
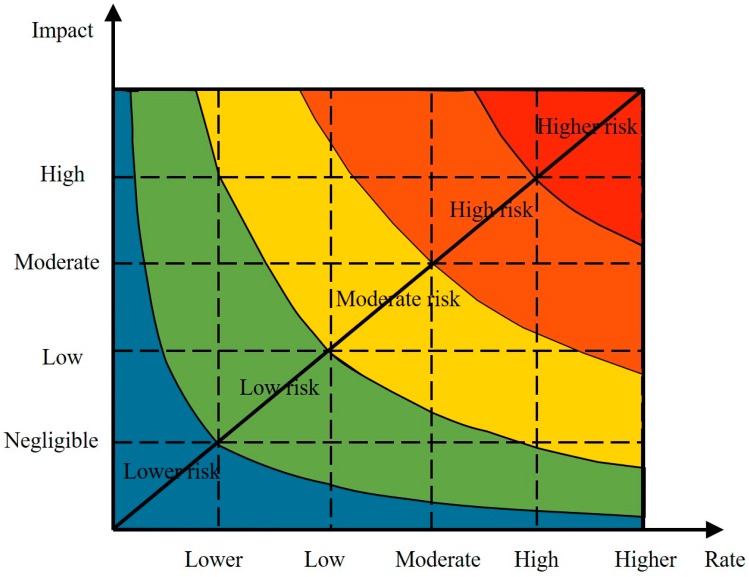
Risk probability—impact matrix schematic.

The risk level refers to certain conditions and within a certain period of time, due to the uncertainty of the results leading to the loss of the behavior of the main body size and the possibility of the loss of size. For the risk probability, the impact of the matrix method was mainly used to analyze the main risk factors of the project and determine the grades of the major risk factors (Figure 4 and Table 3).

According to the *Provisional Measures for Risk Assessment*, the social security risk rating of major projects was divided into three levels [67], and the reference standards of various risk ratings are shown in Table 4.

Single assessments were made for the four types of risk in the project. In order to measure the overall risk of the project easily, this study quantified the possibility of each kind of risk occurring and then determined the comprehensive risk of the project. The method for calculating comprehensive risk is as follows:

(1) R=W∗C

where W refers to the weights of various risk factors. It is determined based on the results of the questionnaire. C represents the risk probability level. This study divided the risk rating into five grades.

The risk events at each stage of the project take different forms, including leaflets, letters and visits, parades, traffic congestion, and the involvement of government departments. The risk consequences caused by all kinds of risk events and the impacts of risk levels are different [67]. According to the impact and stability of the affected cities and surrounding cities, the classifications are shown in Table 5.

## 4. Results

### 4.1. Initial Risk Rating of the Project

In terms of risk factors based on various factors, the initial risk level of the project was low risk (0.32 < 0.36). The comprehensive risk index of the project was 0.32 (Table 6).

### 4.2. Resolution Measures

In view of the above unfavorable risk factors that may cause social instability, the following countermeasures and measures were suggested:

(1) Risk caused by land expropriation standards and the high expectations of the masses

Strictly follow the requirements of the implementation of the standard of compensation for land expropriation, communicate well with the affected people, strengthen education, carry out advocacy measure, and remove the contradiction.

(2) Risk caused by land ownership disputes or uncertain landlords

Land ownership is unclear and should proceed from reality and use facts to maintain the principle of objective and fair treatment. The handling of work must be strictly in accordance with the laws and regulations of the process.

(3) Risk caused by land expropriation compensation not being released in a timely manner

The compensation fee for land expropriation must be paid in full within three months as of the date of approval of the compensation and the resettlement plan for land expropriation; it cannot be paid in installments. It is strictly forbidden to depose, encroach, retain, or misappropriate other land use rights to ensure the timely payment of compensation fees for land expropriation.

(4) Risk caused by compensation for the violation of temporary planting of young crops or ground attachments with the high expectations of the masses

According to the relevant state-owned policies, before land expropriation is submitted for approval, according to law, the local land and resources department must inform the affected rural collective economic organizations and relevant farmers of the requisitioned land use contents, locations, compensation rates, and resettlement methods in written form. After informing, all non-violating temporary planting of young crops or temporarily attached ground attachments shall not be compensated for when they are requisitioned. Relevant functional departments should do a good job in education, publicity, and interpretation before land expropriation to ensure early prevention.

### 4.3. Risk Level after the Measure

After implementing various risk prevention and mitigation measures, the number of stakeholders adversely affected by the project may be reduced, the project’s compatibility with the local community seems like enhanced, and the number of risk events that may arise also seems like reduced. After the measures are implemented, the risk level of the project will be reduced ostensibly.

### 4.4. Assessment Results

Regarding the questionnaire conducted on the surrounding people and the issuance of opinions and suggestions, none of the respondents raised objections about the land expropriation project of Jinsong Old-age Care Services Center in Dingzhou City, Hebei Province.

- The project complies with the provisions of the Land Administration Law of the People’s Republic of China and the Urban and Rural Planning Law of the People’s Republic of China, and it is in line with the overall urban planning and overall land use planning of Dingzhou City.
- The land expropriation and compensation of this project has strictly implemented the relevant compensation standards of Dingzhou City, Hebei Province.
- A land expropriation stability risk assessment was carried out in an orderly manner, with publicity in place. The assessment was in line with relevant laws and regulations as well as local reality and the interests of the masses.
- Using the comprehensive assessment of the risk index, the project’s risk index was calculated to be 0.32. According to the standard of risk classification, the initial risk level of the project is Low (Normal negative impact).
- The social stability risk assessment of land expropriation identified that the overall risk level of the land expropriation of the land parcel is low risk, and the project will be implemented in the context of the related preplan and the resettlement compensation plan.

## 5. Discussion

### 5.1. The Main Risks and Causes of Land Expropriation

The main risk with land expropriation is that the procedure of land expropriation is not standardized. For example, land acquisition projects do not fully comply with land acquisition procedures, and land compensation costs cannot be paid to farmers [63]. The abuse of power by land expropriators will also lead to the risk of land expropriation. For example, most remote rural areas still carry out the tradition of burying. The location of a funeral is often in farmland that has been cultivated for generations. Once the farmland has been expropriated, the construction team often enters the farmland without the consent of the farmer and only with the permission of the government unilaterally. They are “stationed” in the farmland to carry out preliminary construction, for example, the construction of walls and fences. For other land to be expropriated, they draw a circle on the ground and occupy it. These behaviors will be regarded by farmers as disrespectful to their ancestors, so land expropriators have contradictions and can even cause conflicts. Besides, on the land about to be expropriated, crops may be about to mature and harvest, but the construction team may not wait for the farmers to harvest and blindly “catch up” the crops. Farmers will cause conflict because of the destruction of the crops on which they depend, resulting in some villagers blocking the construction of land expropriation.

In view of the resistant behavior of farmers caused by the above factors, land expropriators have taken stable risk reduction measures in order to reduce social risks and maintain social stability. This mainly includes the following measures:

- Strengthening propaganda and advocating the significance and legitimacy of Land expropriation;
- Issuing land expropriation process, compensation procedure standards, and personnel placement policies;
- Early investigation of possible risks arising from land expropriation;
- Local justice, community letters and visits. Village committees coordinate one by one to resolve the risk of land expropriation.

### 5.2. Characteristics of the Land Expropriation Risk Assessment System

Since the reform and opening up over more than 30 years, with the development of socialist democracy and laws, Chinese land expropriation procedures have been improved and the procedures for land expropriation have been strengthened. We found that the current social risk assessment framework is a good way to evaluate the risk of land expropriation projects. Nevertheless, there are still outstanding problems. As a result of land expropriation disputes, illegal land use has accounted for more than 70% of the total petition cases. Of the Chinese Court Cases in 2015, 35,726 cases were related to land acquisition [68].

At the level of laws and regulations, the issue of “attaching importance to substance but neglecting procedure” has not fundamentally changed, and the guarantee of farmers’ right to know, participate in, and supervise the land requisitioned is not enough [66]. For example, we found that the provisions of the law only concern the investigation of land expropriation, focusing on the present situation of utilization and the ownership of property rights, geological risks, and other natural and economic conditions. However, they lack social risk investigation on land expropriation implementation. Moreover, the provisions of the law concerning the enforcement of land expropriation reinforce the obligations of collective economic organizations and farmers but lack coordination and adjudication of disputes over compensation and placement.

From the execution level of laws and regulations, non-standard and improper execution problems are quite common, and quite a large part of land expropriation gives rise to the selective execution phenomenon. For example, farmers who have their land requisitioned do not have sufficient participation in land expropriation and confirmation and hearing procedures are seldom strictly implemented. Most of them only confirm the results of the investigation in advance, understand the compensation standards and resettlement schemes for land expropriation in advance, and have no substantial impact on the decision to carry out land expropriation. The announcement of land expropriation has detailed provisions on the time and content of land expropriation and the time and content of the compensation and resettlement scheme of land expropriation, but the actual implementation is insufficient.

**Figure 5:**
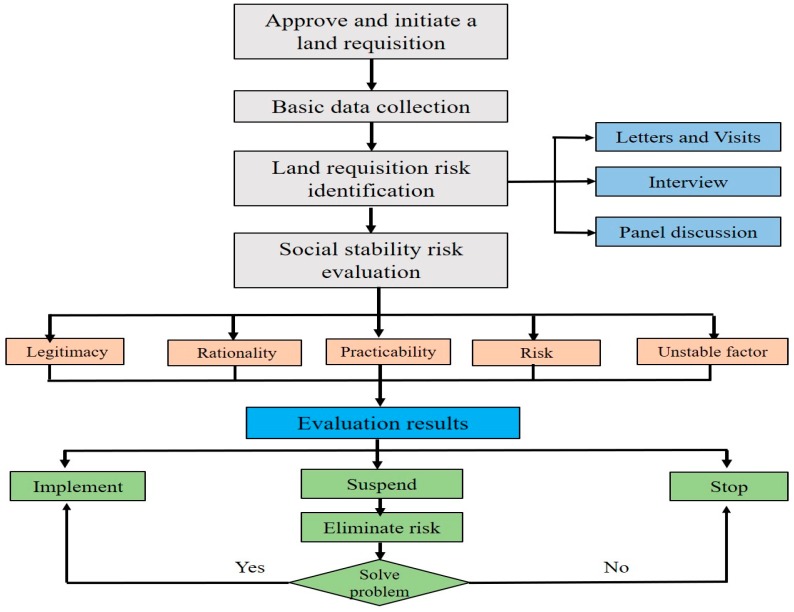
The procedures of the land expropriation risk assessment system in China [69].

In order to forecast the contradictions, disputes, and potential risks caused by land expropriation, the social stability risk assessment system of land expropriation carries out a comprehensive analysis and assessment of projects related to land expropriation or agricultural land conversion before administrative examination and approval [69]. The current land expropriation risk assessment system reflects the willing of landless masses and evaluates social risks and potential problems. “Risk identification” and “Risk reduction” are key components of a social stability risk assessment system, and comprehensive risk is the result of the risk reduction through the social stability risk assessment system. The current social stability risk assessment system is thus a means of maintaining social stability [70]. Therefore, maintaining social stability is the core of the system rather than risk (Figure 5).

The disadvantage of the current social stability risk assessment system for land expropriation is that even if the land expropriation project draws a low risk conclusion through the evaluation, it contains risk factors and both the land expropriator and the expropriated farmers know it contains risks. Even though the risks have not been completely solved, the land expropriation project is implemented hastily. As a result, the land expropriation party invests significant manpower and funds to maintain the stability of the land expropriation project in the later stage of the land expropriation project. Therefore, there are some deficiencies in the current system. The assessment responsibility of the current land expropriation risk assessment system is unclear. The function orientation of the current land expropriation risk assessment system is ambiguous. The procedure of the current land expropriation risk assessment system is nonstandard.

### 5.3. A Comparison between the Present Study and Previous Studies

To examined social stability risks caused by rural land acquisition in China, Wang et al. (2014) investigated 151 administrative villages in 15 provinces (including Jiangsu, Beijing, Liaoning, Shandong, Guangdong, Hebei, Hunan, Henan, Hubei, Anhui, Jilin, Yunnan, Guangxi, Sichuan and Gansu) have been undergoing land expropriation. Their research subjects include farmers who have been working at home for a long time, migrant workers who have returned to their hometowns, and self-employed households with non-agricultural income in their hometowns, which ensure the extensiveness of the research samples. Their results showed that the value of social stability risk index in current rural land expropriation has been reached to 0.68, and it belonged to high (significant negative impact) social risks in term of social stability risk rating criteria. In particular, they found there are different social stability risks among east region, central region and west region of China, However, the risks caused by land acquisition are not much different, and they are all at a high (significant negative impact) social risk level [47] (Table 7).

Comparing with the previous study, our research findings confirm that the risk assessment system of land expropriation in China is aimed at maintaining social stability [70]. It contains preparatory procedures for reducing social risk. After any high risk land expropriation project is evaluated, it can be concluded that, on the one hand, there are risk factors, or on the other hand, the risk is very low. This determines whether or not the preparatory procedure for reducing social risk is implemented. The advantage of the current social stability risk assessment system for land expropriation is that it can reduce the waiting time for land expropriation projects. Land expropriation projects will not be stranded or even delayed because of the high risk level.

## 6. Conclusions and Policy Implications

### 6.1. Conclusions

Based on a case study of land expropriation social stability risk assessment in China, this study investigated the social stability risk caused by land expropriation. The current social stability risk assessment system of land expropriation was analyzed. The results showed that: (1) the current land expropriation risk assessment system mostly considers the land administration department to be the main body of responsibility, identifies the risk by means of seminars, letters and visits, and takes the opinion of the masses or experts as the risk assessment result. In the course of practice, there is an unclear appraisal subject, unclear functional orientation, and unscientific assessment process, among other issues. (2) The current land expropriation risk assessment system should be standardized in terms of defining the risk assessment of land expropriation, improving the land expropriation risk assessment system, and optimizing the land expropriation assessment procedures. To ensure that risk assessment is carried out at the time of construction project establishment and that a scientific and objective assessment method is adopted to establish a risk assessment system that includes land expropriation risk assessment, demolition risk assessment, and environmental impact assessment, the main role of local governments in the risk assessment must be clarified and the land expropriation conflict assessment of supporting mechanisms and regulatory mechanisms must be established and improved.

### 6.2. Policy Implications

Based on the above analysis, we think that the reform of the land expropriation procedure should focus on the following aspects:

(1) Establishment of a land expropriation pre-announcement system: The county government is responsible for drawing up the land expropriation plan. The preannouncement should be made in the townships (towns) and villages where the land is to be expropriated by means of land use, compensation standards, resettlement channels, and social security measures. As the main body of land expropriation, the government should fully listen to the opinions of the members of the collective rural economic organizations and sign agreements with the vast majority of the members of the collective economic organizations or the farmers. Land expropriation can only be initiated after land expropriation compensation and social security charges are implemented.
(2) Establishing the system of confirming the results of land expropriation investigation: After the announcement of the proposed expropriation of land is issued, the local administrative organ should, together with the village collective economic organizations, jointly carry out the site investigation of the land expropriation, and conduct field investigation on the title, category, area, title, type, quantity, etc. of the land to be expropriated. Finally, it should be confirmed by the collective economic organizations, farmers, and property owners of the land expropriated rural areas.
(3) Perfecting the measures of land expropriation and conflict settlement: The government should give full attention to the functions of administrative mediation, administrative reconsideration, administrative litigation, and judicial arbitration to prevent and resolve contradictions and disputes over land expropriation. On the other hand, the government should clarify ways to resolve the differences in land expropriation and compensation agreements within collective rural economic organizations.
(4) Standardizing the publicity of land expropriation Information: Land expropriation approval documents, scope, compensation, resettlement, and other information should be unified and made public by the county government and should be subject to supervision by the masses and society. In addition to the confidential content, the relevant land expropriation information should be made public.

## Figures and Tables

**Table 1:** Some main questions for the questionnaire.

Inquiries	Specific Items/Questions
Personal attributes	Name, occupation, residency
Opinions, Concerns, Measures/suggestions	How do you know this project?
	Do you think the project will have a negative impact on you?
	What adverse effects do you think the project will have on you?
	If the project has an impact on you, are you willing to accept it?
	Do you support the implementation of this project?
	If you object to the implementation of the project, please give reasons.
	Do you have any suggestions for this project?

**Table 2:** Social stability risk factor identification table [67].

	Number	Risk Factors	Risk Factor Content
Land expropriation and house demolition and compensation	1	Land expropriation and house demolition and compensation	Whether the construction land is in line with the local conditions, the overall requirements of saving and using the land resources, the relationship between the land expropriation scope, and the land use planning
	2	Land expropriation compensation funds	Source of funds, quantity, implementation
	3	Landless peasants’ re-employment and living	Peasant society, health insurance programs and implementation, skills training, and employment programs
	4	Settlement availability and quality	The total housing ratio, the proportion of regional housing, the existing ratio, the availability of housing, and the planning level of support and the degree of integration
	5	Land expropriation compensation standards	The relationship between physical or monetary compensation and the market price and the recent similar land compensation standards (too much or too little are not reasonable)
	6	Land expropriation and compensation procedures and programs	Whether to carry out the compensation for land expropriation according to the procedures stipulated by national and local laws and regulations; whether the compensation plan solicits public opinions, etc.
	7	Demolition process	Supervision of the formulation and demolition of civilized demolition programs, past performance and impact of demolitions, etc.
	8	Special land and building expropriation	Involves basic farmland expropriation and expropriation, military land, religious land expropriation and expropriation, whether there are connections with the relevant policies, etc.
	9	Pipeline relocation and green relocation program	Pipeline relocation and afforestation relocation plan rationality
	10	Other types of compensation	Compensation scheme for buildings damaged by construction, compensation scheme for people receiving various types of living environment due to project implementation, etc.

**Table 3:** Main risk assessment of the project.

Number	Risk Factors	Risk Impact	Risk Rate	Level of Risk
1	Risks caused by land expropriation standards and the high expectations of the masses	Moderate	Moderate	Normal
2	Risks caused by land ownership disputes or uncertain landlords	Moderate	Moderate	Normal
3	Risks caused by land expropriation compensation, not timely release	Moderate	Moderate	Normal
4	Risks caused by compensation for the violation of temporary planting of young crops or ground attachments with the high expectations of the masses	Moderate	Moderate	Normal

**Table 4:** Social stability risk rating criteria for reference.

Level of Risk	High (Significant Negative Impact)	Moderate (High Negative Impact)	Low (Normal Negative Impact)
General assessment criteria	Most people have an objection or a different opinion on the project, i.e., they reflect particularly strongly. This may cause large-scale mass events	Some people have an objection or a different opinion on the project, i.e., they reflect strongly. This may cause conflict	Most of the masses understand and support the project, but a small number of people have an objection or a different opinion on the project. Contradictions can be prevented and resolved through effective work
Criteria for possible risk events	Such as the attack of party and government organizations, key departments, and key areas; hitting, smashing, robbing, killing, and other types of collective fighting; mob riots; casualties; illegal rallies; demonstrations, processions; strikes; etc.	Such as collective petitions, the occurrence of extreme personal events, containment sites, blocking, blocking traffic, etc.	Such as individual abnormal petitions, sitting, pulling banners, shouting slogans, distributing promotional materials, etc.
Criteria for the number of participants in risk events	More than 200 people	20–200 people	Less than 20 people
Single factor risk level assessment criteria	2 or more significant risk factors or 5 high single risk factors	1 significant risk factor or 2–4 high single risk factors	1 high or 1–4 normal single risk factors
Comprehensive risk index for assessment criteria	>0.64	0.36–0.64	<0.36

**Table 5:** Risk event and risk consequence assessment form.

Risk Consequences	Higher Risk	High Risk	Normal Risk	Possibility of the Issue
The assailant explodes and sets himself on fire	√			Generally, does not happen
A government department is attacked	√			Generally, does not happen
Traffic is blocked	√			Generally, does not happen
The project construction site is attacked		√		Less likely to occur
Collective petitions are formed		√		Less likely to occur
There is a parade to protest		√		It may happen
Information is published on the web		√		It may happen
There is a hanging banner protest			√	It may happen
Dissemination CD/leaflet protest			√	It may happen
A petition is written			√	It may happen

**Table 6:** Comprehensive risk index quantitative calculation table.

Main Risk Factors	Risk Weight	Risk Level					Risk Index W*C
		Lower	Low	Moderate	High	Higher	
		0.04	0.16	0.36	0.64	1	
Risk caused by land expropriation standards and the high expectations of the masses	0.24			√			0.0864
Risk caused by land ownership disputes or uncertain landlords	0.26			√			0.0936
Risk caused by land expropriation compensation not released in a timely manner	0.2			√			0.032
Risk caused by compensation for the violation of the temporary planting of young crops or ground attachments with the high expectations of the masses	0.3			√			0.108
ΣW*C	1						0.32

**Table 7:** Social risk assessment results in China [47].

Region	Risk Index	Grade
East	0.621	High (significant negative impact)
Central	0.689	High (significant negative impact)
West	0.662	High (significant negative impact)
Total	0.677	High (significant negative impact)

Note: East region includes Jiangsu, Beijing, Liaoning, Shandong, Guangdong, Hebei; Central region includes Hunan, Henan, Hubei, Anhui, Jilin; West region includes Yunnan, Guangxi, Sichuan and Gansu.
